# Ternary Fingerprints with Reference Odor for Fluctuation-Enhanced Sensing

**DOI:** 10.3390/bios10080093

**Published:** 2020-08-09

**Authors:** Xiaoyu Yu, Laszlo B. Kish, Jean-Luc Seguin, Maria D. King

**Affiliations:** 1 Department of Biological and Agricultural Engineering, Texas A&M University, College Station, TX 77843-2117, USA; yuxy69@mail.sysu.edu.cn; 2 School of Atmospheric Sciences, Sun Yat-sen University, Zhuhai 519000, China; Laszlokish@tamu.edu; 3 Department of Electrical and Computer Engineering, Texas A&M University, College Station, TX 77843-3128, USA; j-l.seguin@univ-amu.fr

**Keywords:** fluctuation enhanced sensing, cow manure bacteria, odor sensing, trinary fingerprints

## Abstract

An improved method for fluctuation-enhanced sensing (FES) is introduced. We enhanced the old binary fingerprinting method, where the fingerprint bit values were ±1, by introducing ternary fingerprint bits utilizing a reference odor. In the ternary method, the fingerprint bit values are −1, 0, and +1, where the 0 value stands for the situation where the slope of the spectrum is identical to that of the reference odor. The application of the reference odor spectrum makes the fingerprint relative to the reference. The ternary nature and the reference feature increase the information entropy of the fingerprints. The method is briefly illustrated by sensing bacterial odor in cow manure isolates.

## 1. Introduction: Fluctuation-Enhanced Sensing (FES)

Fluctuation-enhanced sensing (FES) [1,2,3,4,5,6,7,8,9,10,11,12,13,14,15,16,17,18,19,20,21,22,23,24,25,26,27,28,29,30,31,32,33,34,35,36,37,38,39,40,41] evolved from the observations that the random fluctuations of physical quantities potentially carry more information about the physical system than their average value. This statement is also valid for sensory signals and conductance noise of samples with non-passivated surfaces indicated an unwelcome external interference in laboratory experiments.

**Figure 1:**
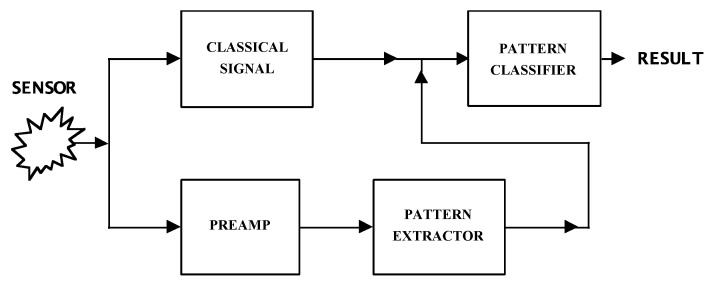
Fluctuation-enhanced sensing scheme.

The FES method utilizes the statistical properties of microscopic random fluctuations superimposed on the classical sensor signal to generate patterns that identify the chemical composition of odors (see Figure 1). The classical signal is often a DC voltage value (due to the sensor resistance response); the pattern extractor is often a spectrum analyzer generating the power density spectrum of the pre-amplified sensor noise voltage; and the pattern classifier is often a neural network, or more advanced methods as described in the text. 

For the non-specialist reader, we give a very brief history of FES developments below which is centered about the related patents.

In 1994–1995, electrical noise for sensing chemicals was proposed by showing the variations of conductance noise spectra of conducting polymers as a function of the ambient gas composition [1,2]. In 1997, similar observations were made about the conductance noise spectrum of semiconductor resistors with non-passivated surfaces [3]. 

The first patented FES scheme [4] appeared in 1998 and it was followed by a number of patents [5,6,7,8,9,10] during the subsequent years. 

The first analytic scheme of a generic FES systems for quantitative analysis of gas mixtures with mathematical analysis about the limits and with the sensor number requirement versus the number of agents [4,11]. 

The possibility of “freezing the odor”, that is, the sampling-and-hold technique [5,12] in a Taguchi gas sensor, was an improvement with memory and robustness of gas turbulences due to heat convection. 

The spectrum of surface diffusion noise on surface acoustic wave (SAW) sensors and open-gate MOS sensors was utilized for another FES technique [6,7,13,14].

The use of higher-order statistics and higher-order spectra was applied to enhance the extracted information from the stochastic signal component [8,15,16].

In a water-based medium, to detect and identify bacteria promptly by FES, the bacteriophage-based microscopy electrochemical cells were proposed and demonstrated [9,10,17,18]. 

The FES technique has been tested by a large body of investigations in many different systems and various conditions, see for example [19,20,21,22,23,24,25,26,27,28,29,30,31,32,33,34,35,36,37,38,39,40,41] and the present paper is a part of the ongoing research and developments to further explore and develop this method.

It is important to note that the pattern generation/recognition aspects are the subject of intensive research that goes beyond the scope of our papers—see for example [42,43,44,45]. Obviously, such research can incorporate artificial intelligence-related technologies, too. Note, these technologies are data processing-intensive, and thus require higher energy dissipation than the simple fingerprinting ideas shown below.

## 2. Materials and Methods

### 2.1. Binary Fingerprints

In principle, the generated patterns, such as the power density spectra (PDS), bispectra, etc., can directly be fed into a classifier (neural network and other machine learning/artificial intelligence tools [42,43,44,45]), to identify the chemical composition related to this pattern. However, machine learning tools require intensive data processing which implies a large energy dissipation. Moreover, the training of machine learning and neural networks is a tremendous task, as it requires executing a great number of measurements with a large variety of chemical compositions.

**Figure 2:**
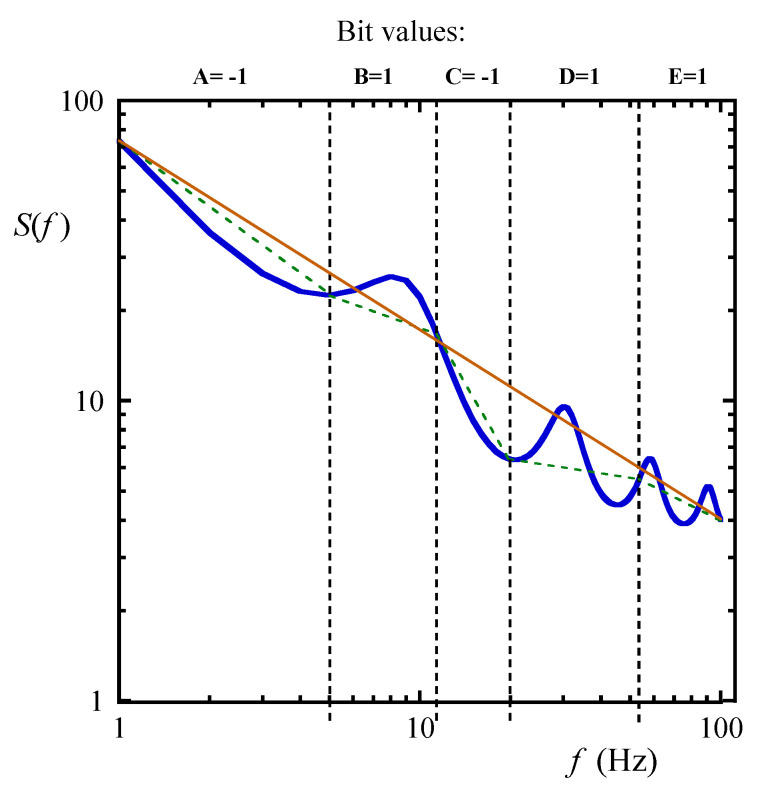
Generating the binary fingerprints [23]. The local slope (green dashed lines) of the spectrum (blue solid line) is compared with the global slope (red solid line). The bit values are −1 (bits A, C) and 1 (bits B, D, E) depending on the result of this comparison.

**Figure 3:**
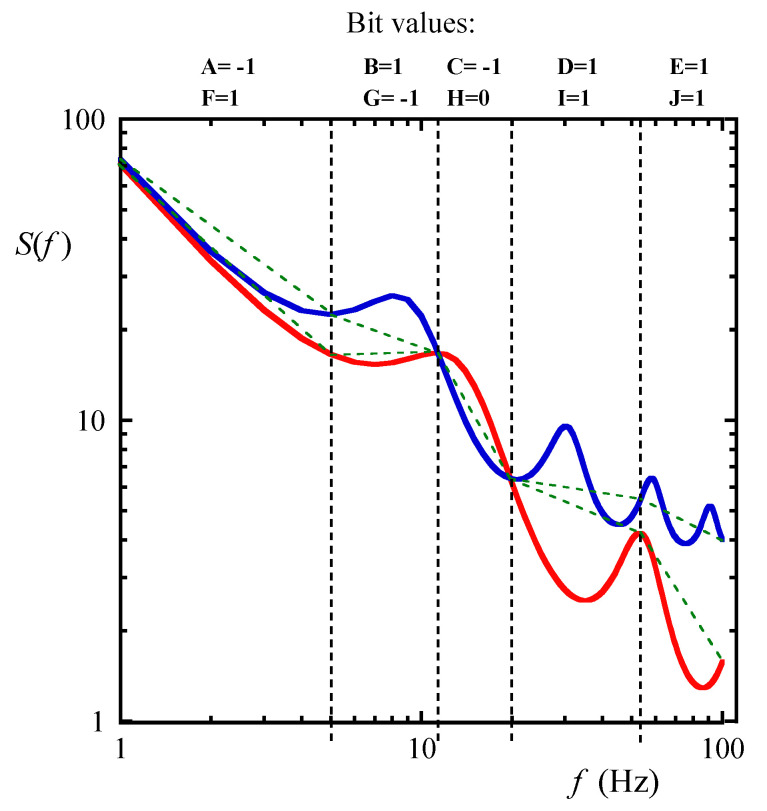
Generating the ternary fingerprints of the same PDS (blue solid line). The reference spectrum is shown with the red solid line. When the local slope of the spectrum (green dashed lines) is greater than the local slope of the reference spectrum (green dashed lines), the bit value is 1 (bits F, I, J). In the opposite case it is −1 (bit G). When the slopes are equal, the bit value is zero (bit H). The binary fingerprints of the PDS are also shown (bits A–E).

Thus, much simpler and more direct approaches have also been tested with good results, for example the binary fingerprint method that extracts a bit string from the measured PDS [46] (see Figure 2). By generating a bit pattern characterizing the chemical environment, this bit pattern can be used as an address that directly calls the name of the chemical environment which requires only a miniscule energy dissipation. The average slope of the spectrum plotted with a log-log scale is determined by connecting the beginning and the end of the (“meaningful” part of the) PDS. Next, the same frequency band is divided into sub-bands to determine the related binary bit values. Then the local slope over these sub-bands is determined in the same way as described above. When the local slope is below the average, the bit value is −1, and otherwise it is 1 (see Figure 3).

As soon as we have such a bit sequence, which is a binary fingerprint to characterize a chemical environment, we need only a simple interpreter to display the name of this chemical environment. Such a system can be useful in simple applications where the low power consumption is also an issue.

In the present paper, we generalized the binary method to the ternary one, where each fingerprint bit had three alternative values instead of two. The new method offers additional information and ways to use comparative features with reference odors. After introducing the ternary method, we demonstrate and compare it with the binary one by generating these fingerprints with cow-manure related odor. For simplicity but without limiting generality, we are talking about PDS as a source pattern, but any other quasi-continuum patterns are suitable.

### 2.2. The New Method: Ternary Fingerprints

In computer science, it has been well-known and demonstrated [47] that a computer with ternary bits having three different values instead of the usual binary bits of just two values is much more powerful than the binary computer version. A ternary bit has ln(3)/ln(2) times higher information entropy, which is about a 50% increase compared to a binary bit, and it has advantages in the processing, too.

Moreover, for the ternary fingerprint method, we also include an enhancement by a reference agent that further increases its potential information content because various different ternary fingerprints can be generated about the same chemical environment by using alternative references. The spectrum of the reference agent serves as the reference PDS. Next, the frequency band is divided into sub-bands (similarly to the case of the binary fingerprints) to determine the related ternary bit values. Then, the local slope on these sub-bands is determined in the same way as described above.

**Figure 4:**
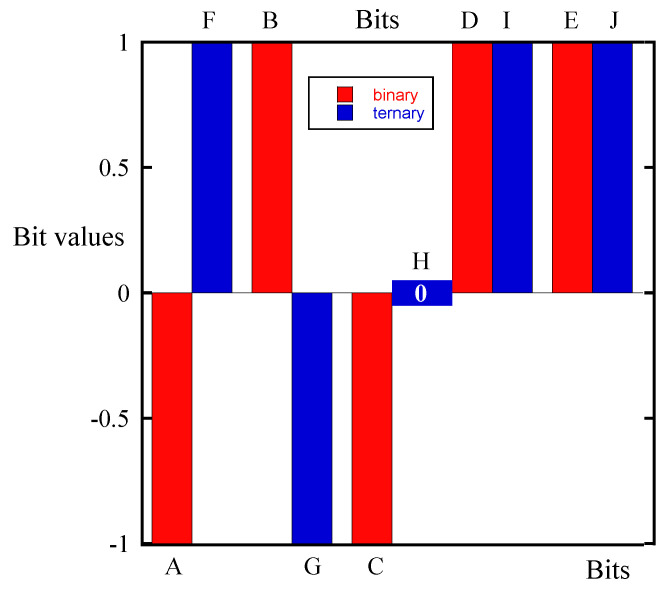
Joint plot of the binary and ternary fingerprints based on the source spectra shown in Figure 2 and Figure 3.

When the local slope with the agent is less than the local slope with the reference agent, the bit value is −1; when it is greater than the reference slope, the bit value is 1; and when the slopes are equal (this happens with a small probability depending on the resolution), the bit value is zero, as shown in Figure 3 and Figure 4.

### 2.3. Demonstration with Bacterial Isolates from Cow Manure

For the demonstration of the ternary fingerprinting method introduced above, we used a bacterial strain isolated from cow manure. The Petri plates of 58 cm^2^ with the bacteria colonies were placed in a 300 cm^3^ sensor chamber with the sensors attached. 

The microorganism used in this study was a Gram-positive toxin producing, facultative anaerobic bacteria, *Bacillus cereus*. Mid-log phase (OD_600_ = 0.5, optical density at 600 nm) cultures of *Bacillus cereus*, isolated from cow manure at a dairy center in Stephenville, Texas were grown in Luria Bertani (LB) medium [1] for 4 h at 37 °C and at 150 rpm. The most abundant bacterium was isolated from different manure samples and identified as *Bacillus cereus* by whole genome sequencing at TIGSS (Texas A&M University Institute for Genome Sequencing and Society). One hundred microliters of the *B. cereus* culture were spread on Difco tryptic soy agar (TSA) plates (Becton Dickinson Co., Sparks, MD 21152, USA), and the plates were incubated overnight at 37 °C [48]. As a reference, sterile TSA plates without bacteria were also prepared. As the TSA medium itself has a strong smell, identical amounts (27 mL) of TSA medium were poured into each plastic Petri plate (VWR, Bridgeport, NJ, USA) to maintain a constant level of background odor [48].

The metal oxide (Taguchi type) sensor was a 50 nm thick SnO_2_ film, sputtered on a 4 × 4 µm^2^ microhotplate with a platinum heater and sensing electrodes. It was designed and manufactured by IM2NP laboratory and details were described elsewhere [49].

## 3. Results and Discussion

**Figure 5:**
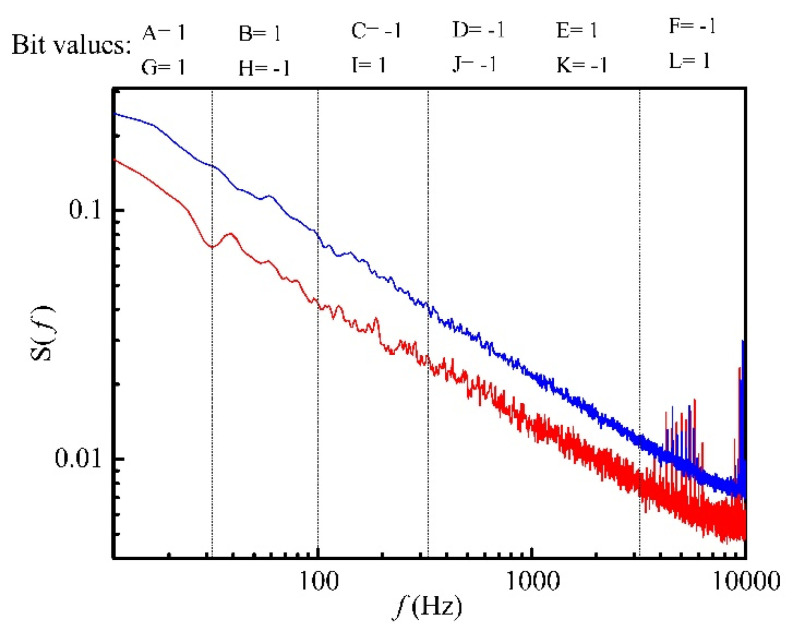
The PDS of the bacterium isolate measured by the Taguchi sensor [49] shown by the blue line. For the reference spectrum, the PDS was measured in laboratory air, shown by the red line.

**Figure 6:**
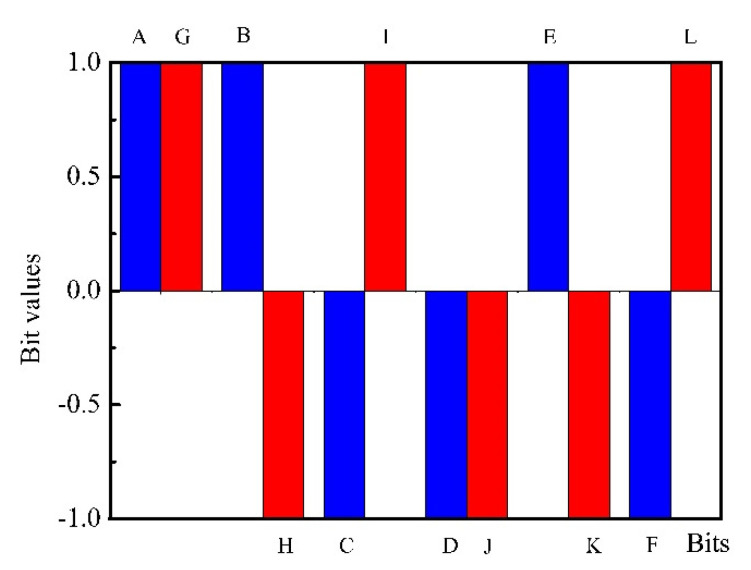
Binary and ternary fingerprints of the bacterium sample. Red: measurement-1; blue: measurement-2 (of the same sample).

Figure 5 shows the measured PDS with the bacterial sample and the reference PDS which was measured using the same sensor in laboratory air. Figure 6 shows the binary and ternary fingerprints extracted from the spectra shown in Figure 5, respectively. 

**Figure 7:**
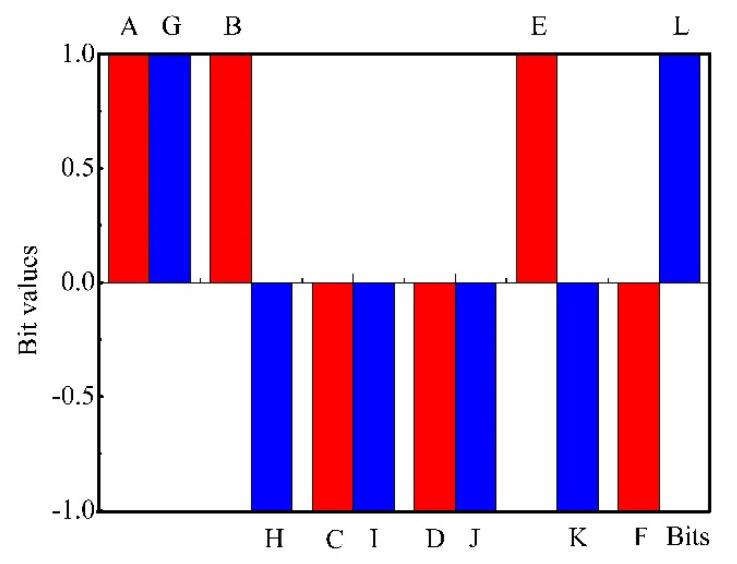
Reproducibility of the binary fingerprints. Red: measurement-1; blue: measurement-2 (of the same sample).

The reproducibility of the odor sensing systems is of great importance. Formerly binary spectra showed a good reproducibility with *Escherichia coli* and *Bacillus anthracis* (anthrax) bacterial samples [23]. Similarly, the reproducibility of the binary fingerprints for the manure isolate *Bacillus cereus* was satisfactory (Figure 7). Therefore, with the ternary fingerprint method, a good reproducibility was also expected.

**Figure 8:**
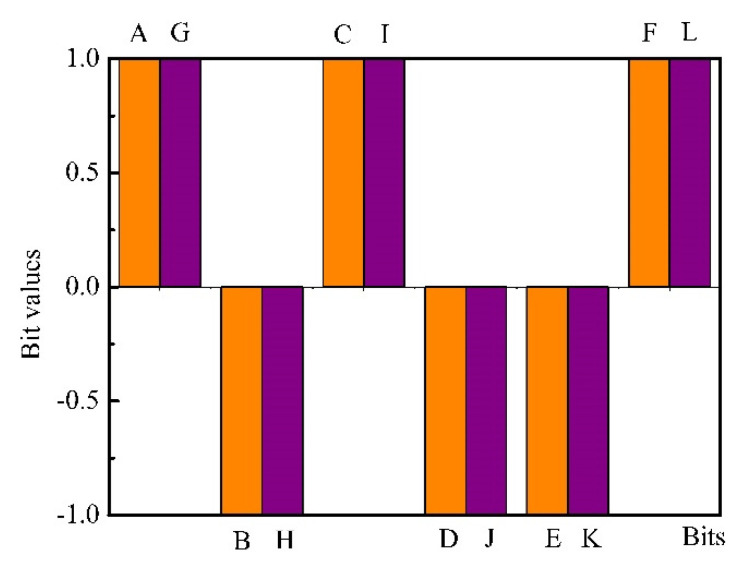
Reproducibility of the ternary fingerprints. Orange: measurement-1; maroon: measurement-2 (of the same sample).

We tested the reproducibility of the ternary fingerprints with our sensor system and bacteria and reference samples. Figure 8 indicates that the reproducibility results of the ternary fingerprints are satisfactory. 

## 4. Conclusions

An improved method for evaluating fluctuation-enhanced sensing (FES) results was introduced. In the ternary method, the fingerprint bit values are −1, 0, and +1, where the 0 value stands for the situation where the slope of the spectrum is identical to that of the reference odor. This step increases the information entropy of the bit pattern by 50%. The application of the reference odor spectrum makes the fingerprint relative to the reference, which further increases the information entropy of the fingerprints.

Measuring bacterial odor in cow manure isolates indicates good reproducibility.

Bit-based direct fingerprinting methods like this one are less intelligent than machine learning tools [42,43,44,45], but they have reduced energy dissipation and can be important in specific FES applications.

## Data Availability

The data that support the findings of this study are available on request from the corresponding author, M.D.K.

## References

[1] Bruschi P., Cacialli F., Nannini A., Neri B. (1994). Gas and vapour effects on the resistance fluctuation spectra of conducting polymer thin-film resistors. Sens. Actuators B Chem..

[2] Bruschi P., Nannini A., Neri B. (1995). Vapour and gas sensing by noise measurements on polymeric balanced bridge microstructures. Sens. Actuators B Chem..

[3] Gottwald P., Kincses Z., Szentpali B., Doering C.R., Kiss L.B., Shlesinger M.F. (1997). Unsolved Problems of Noise (UPoN’96).

[4] Kiss L.B., Granqvist C.G., Söderlund J. Detection of Chemicals Based on Resistance Fluctuation-Spectroscopy. http://was.prv.se/spd/pdf/8V-xToJGAh7WS3oljenFlQ/SE513148.C2.pdf.

[5] Kish L.B., Granqvist C.G., Vajtai R. Sampling-and-Hold Chemical Sensing by Noise Measurements for Electronic. http://was.prv.se/spd/pdf/RdizounvzhfWS3oljenFlQ/SE515249.C2.pdf.

[6] Schmera G., Kish L.B. (2007). System and Method of Fluctuation Enhanced Gas-Sensing Using Saw Devices. U.S. Patent.

[7] Schmera G., Kish L.B. (2009). System and Method of Molecule Counting Using Fluctuation Enhanced Sensors. U.S. Patent.

[8] Smulko J., Kish L.B., Schmera G. (2010). System and Method for Gas Recognition by Analysis of Bispectrum Function. U.S. Patent.

[9] Kish L.B., King M.D., Young R., Cheng M., Biard J.R., Bezrukov S. (2007). Sensing Phage-Triggered Ion Cascade (SEPTIC). U.S. Patent.

[10] Schmera G., Kish L.B. (2017). Bacteria Identification by Phage Induced Impedance Fluctuation Analysis, BIPIF. U.S. Patent.

[11] Kish L.B., Vajtai R., Granqvist C.-G. (2000). Extracting information from noise spectra of chemical sensors: Single sensor electronic noses and tongues. Sens. Actuators B.

[12] Solis J.L., Kish L.B., Vajtai R., Granqvist C.G., Olsson J., Schnurer J., Lantto V. (2001). Identifying natural and artificial odors through noise analysis with a sampling-and-hold electronic nose. Sens. Actuators B.

[13] Schmera G., Kish L.B. (2002). Fluctuation Enhanced Chemical Sensing by Surface Acoustic Wave Devices. Fluct. Noise Lett..

[14] Schmera G., Kish L.B. (2003). Surface diffusion enhanced chemical sensing by surface acoustic waves. Sens. Actuators B.

[15] Smulko J., Granqvist C.G., Kish L.B. (2001). On the statistical analysis of noise in chemical sensors and its application for sensing. Fluct. Noise Lett..

[16] Smulko J.M., Kish L.B. (2004). Higher-Order Statistics for Fluctuation-Enhanced Gas-Sensing. Sens. Mater..

[17] Dobozi-King M., Seo S., Kim J.U., Young R., Cheng M., Kish L.B. (2005). Rapid Detection and Identification of Bacteria: SEnsing of Phage-Triggered Ion Cascade (SEPTIC). J. Biol. Phys. Chem..

[18] Kish L.B., Schmera G., King M.D., Cheng M., Young R., Granqvist C.G. (2008). Fluctuation-Enhanced Chemical/Biological Sensing and Prompt Identification of Bacteria by Sensing of Phage Triggered Ion Cascade (SEPTIC). Int. J. High Speed Electron. Syst..

[19] Gomri S., Seguin J.-L., Aguir K. (2005). Modeling on oxygen chemisorption-induced noise in metallic oxide gas sensors. Sens. Actuators B Chem..

[20] Gomri S., Seguin J.-L., Guerin J., Aguir K. (2006). Adsorption–desorption noise in gas sensors: Modelling using Langmuir and Wolkenstein models for adsorption. Sens. Actuators B Chem..

[21] Gomri S., Seguin J.-L., Guerin J., Aguir K. (2008). A mobility and free carriers density fluctuations based model of adsorption–desorption noise in gas sensor. J. Phys. D Appl. Phys..

[22] Contaret T., Seguin J.-L., Menini P., Aguir K. (2012). Physical-based characterization of noise responses in metal-oxide gas sensors. IEEE Sens. J..

[23] Gomri S., Contaret T., Seguin J., Aguir K., Masmoudi M. (2017). Noise modeling in MOX gas sensors. Fluct. Noise Lett..

[24] Gomri S., Contaret T., Seguin J.-L. (2018). A New Gases Identifying Method with MOX Gas Sensors Using Noise Spectroscopy. IEEE Sens. J..

[25] Gomri S., Seguin J., Contaret T., Fiorido T., Aguir K. (2018). A noise spectroscopy-based selective gas sensing with MOX gas sensors. Fluct. Noise Lett..

[26] Gomri S., Bedoui S., Morati N., Fiorido T., Contaret T., Seguin J.-L., Kachouri A., Masmoudi M. (2019). A Noise Spectroscopy-Based Features Extraction Method to Detect Two Gases Using One Single MOX Sensor. IEEE Sens. J..

[27] Hoel A., Ederth J., Kopniczky J., Heszler P., Kish L.B., Olsson E., Granqvist C.G. (2002). Conduction invasion noise in nanoparticle WO_3_/Au thin-film devices for gas sensing application. Smart Mater. Struct..

[28] Solis J.L., Seeton G., Li Y., Kish L.B. (2003). Fluctuation-Enhanced Sensing with Commercial Gas Sensors. Sens. Transducers Mag..

[29] Solis J.L., Seeton G.E., Li Y., Kish L.B. (2005). Fluctuation-Enhanced Multiple-Gas Sensing. IEEE Sens. J..

[30] Kish L.B., Li Y., Solis J.L., Marlow W.H., Vajtai R., Granqvist C.G., Lantto V., Smulko J.M., Schmera G. (2005). Detecting Harmful Gases Using Fluctuation-Enhanced Sensing. IEEE Sens. J..

[31] Smulko J.M., Ederth J., Li Y., Kish L.B., Kennedy M.K., Kruis F.E. (2005). Gas-Sensing by Thermoelectric Voltage Fluctuations in SnO_2_ Nanoparticle Films. Sens. Actuators B.

[32] Ederth J., Smulko J.M., Kish L.B., Heszler P., Granqvist C.G. (2006). Comparison of classical and fluctuation-enhanced gas sensing with PdxWO3 nanoparticle films. Sens. Actuators B.

[33] Kish L.B., Smulko J., Heszler P., Granqvist C.G. (2007). On the sensitivity, selectivity, sensory information, and optimal size of resistive chemical sensors. Nanotechnol. Percept..

[34] Kwan C., Ayhan B., Chen G., Chang C., Wang J., Ji B. (2006). A Novel Approach for Spectral Unmixing, Classification, and Concentration Estimation of Chemical and Biological Agents. IEEE Trans. Geosci. Remote Sens..

[35] Ayhan B., Kwan C., Zhou J., Kish L.B., Benkstein K.D., Rogers P.H., Semancik S. (2013). Fluctuation enhanced sensing (FES) with a nanostructured, semiconducting metal oxide film for gas detection and classification. Sens. Actuators B Chem..

[36] Makra P., Topalian Z., Granqvist C.G., Kish L.B., Kwan C. (2012). Accuracy versus speed in fluctuation-enhanced sensing. Fluct. Noise Lett..

[37] Kish L.B., Chang H.C., King M.D., Kwan C., Jensen J.O., Schmera G., Smulko J., Gingl Z., Granqvist C.G. (2011). Fluctuation-Enhanced Sensing for Biological Agent Detection and Identification. IEEE Nanotechnol..

[38] Gingl Z., Kish L.B., Ayhan B., Kwan C., Granqvist C.-G. (2010). Fluctuation-Enhanced Sensing with Zero-Crossing Analysis for High-Speed and Low-Power Applications. IEEE Sens. J..

[39] Chang H.C., Kish L.B., King M.D., Kwan C. (2009). Fluctuation-Enhanced Sensing of Bacterium Odors. Sens. Actuators B.

[40] Kwan C., Schmera G., Smulko J., Kish L.B., Heszler P., Granqvist C.G. (2008). Advanced agent identification at fluctuation-enhanced sensing. IEEE Sens. J..

[41] Aroutiounian M., Mkhitaryan Z., Adamian A., Granqvist C.-G., Kish L.B. (2009). Fluctuation-enhanced gas sensing. Proced. Chem..

[42] Duan H., Li H., Xie J., Panikov N.S., Cui H.L. (2011). Agent identification using a sparse Bayesian model. IEEE Sens. J..

[43] Wan S., Mak M.W., Kung S.Y. (2012). mGOASVM: Multi-label protein subcellular localization based on gene ontology and support vector machines. BMC Bioinform..

[44] Smulko J.M., Ionescu R., Granqvist C.G., Kish L.B. (2015). Determination of gas mixture components using fluctuation enhanced sensing and the LS-SVM regression algorithm. Metrol. Meas. Syst..

[45] Wan S., Mak M.W., Kung S.Y. (2016). Sparse regressions for predicting and interpreting subcellular localization of multi-label proteins. BMC Bioinform..

[46] Chang H.C., Kish L.B., King M.D., Kwan C. (2010). Binary Fingerprints at Fluctuation-Enhanced Sensing. Sensors.

[47] Glusker M., Hogan D.M., Vass P. (2005). The Ternary Calculating Machine of Thomas Fowler. IEEE Ann. Hist. Comput..

[48] Sambrook J., Fritsch E.F., Maniatis T. (1989). Molecular Cloning: A Laboratory Manual.

[49] Favard A., Yan X., Anguille S., Moulin P., Seguin J.-L., Aguir K., Bendahan M. (2018). Ionic Liquids Filter for Humidity Effect Reduction on Metal Oxide Gas Sensor Response. Sens. Transducers.

